# Endoscopic stricturotomy for axis-related gastric outlet obstruction following sleeve gastrectomy

**DOI:** 10.1055/a-2743-3189

**Published:** 2025-12-03

**Authors:** Luana Gabriela dos Santos, Miriam Chinzon, Alexandre Moraes Bestetti, Diogo Turiani Hourneaux de Moura, Eduardo Guimarães Hourneaux de Moura

**Affiliations:** 1 117265 Graduação, Hospital das Clínicas da Faculdade de Medicina da Universidade de São Paulo, São Paulo, Brazil; 2 Gastrointestinal Endoscopy Unit, Hospital das Clínicas da Faculdade de Medicina da Universidade de São Paulo, Ribeirao Preto, Brazil

**Keywords:** Endoscopy Upper GI Tract, Dilation, injection, stenting, POEM, Endoscopic resection (ESD, EMRc, ...)

## Abstract

**Background and study aims:**

Axis deviation and helical stricture following sleeve gastrectomy may result in functional gastric outlet obstruction, leading to significant patient morbidity. Conventional endoscopic therapies, such as balloon dilation and self-expandable metal stents (SEMS), have demonstrated limited efficacy and are frequently associated with recurrence and complications. This study aimed to assess clinical outcomes of endoscopic stricturotomy as a minimally invasive technique for managing axis-related stenosis after sleeve gastrectomy.

**Patients and methods:**

This retrospective case series included adult patients (≥ 18 years) who underwent endoscopic stricturotomy for axis-related stenosis at a tertiary academic center from 2019 to 2024. Diagnosis of helical stricture was confirmed via endoscopic and radiologic assessments. Clinical data were obtained through electronic chart review and structured telephone interviews. Symptom severity was evaluated using the Gastroparesis Cardinal Symptom Index (GCSI), and quality of life was assessed using a visual analog scale (VAS). Statistical analysis included the Shapiro-Wilk test and paired t-test or Wilcoxon signed-rank test, with *P* < 0.05 considered significant.

**Results:**

Eight patients (mean age 53 years; 62.5% female) were included, with a mean time of 31.3 months between surgery and stricturotomy. All GCSI domains showed significant symptom improvement. The global GCSI score improved from 35.44 to 21.66 ( *P* = 0.014), and VAS scores increased from 3.0 to 7.75. Complications included two cases of intraoperative pneumoperitoneum and one delayed gastric fistula, all managed non-surgically.

**Conclusions:**

Endoscopic stricturotomy is a promising, minimally invasive treatment for axis-related gastric outlet obstruction post-sleeve gastrectomy. Prospective studies are warranted to confirm long-term outcomes.

## Introduction

Obesity has increased continuously in recent decades and, according to recent data from the World Health Organization, approximately 16% of adults had obesity in 2022 (age-standardized prevalence ≈ 13.6% in 2022) 1 . It is associated with a heightened risk of chronic comorbidities, a substantial decline in quality of life (QoL), and a significant economic burden on Brazil’s public healthcare system 2 .

In response to this escalating epidemic, bariatric surgery has emerged as an effective intervention for severe obesity, offering improvements in both clinical outcomes and QoL while simultaneously reducing long-term healthcare expenditures 3 .

Among the various bariatric procedures, sleeve gastrectomy is one of the most frequently performed. It involves resecting approximately 75% to 80% of the stomach, resulting not only in mechanical restriction but also in hormonal modulation, particularly through decreased ghrelin production, a key regulator of appetite and satiety. This procedure is associated with an excess weight loss of approximately 50% to 60% within 2 to 3 years 3 .

Despite its overall efficacy, sleeve gastrectomy is associated with potential adverse events (AEs), most notably postoperative fistulas and strictures. It has been proposed that asymmetric traction or misalignment of the gastric sleeve during surgery may result in rotation and axis deviation. This can create a functional obstruction characterized by rotational angulation of the gastric tube, impairing food passage without a fixed anatomical narrowing. In contrast, true gastric stenosis arises from localized fibrosis and luminal narrowing, representing a structural obstruction. Together, these complications account for functional gastric outflow obstruction in approximately 0.7% to 4% of cases. Clinically, patients may present with progressive dysphagia to solids and liquids, nausea, vomiting, and, in severe cases, malnutrition 4 .

Diagnosis is typically established via upper gastrointestinal endoscopy and contrast radiographic studies of the esophagus, stomach, and duodenum. Management strategies range from revisional surgery to endoscopic interventions. However, conventional endoscopic approaches such as pneumatic balloon dilation and self-expanding metal stents (SEMS) have demonstrated limited long-term efficacy, often necessitating repeated procedures and carrying risks such as stent migration 4 5 .

First described in 2018 6 , endoscopic stricturotomy is an advanced technique that requires specific training and prior experience, and has emerged as a minimally invasive alternative. The technique involves creation of a submucosal tunnel along the staple line, enabling a targeted myotomy to correct torsion and relieve outflow obstruction. By reducing anatomical distortion and enhancing gastric compliance, this approach has shown promise in improving clinical symptoms.

This study presents a case series of patients with post-sleeve gastrectomy strictures treated with endoscopic stricturotomy. The primary outcome was clinical response. Secondary outcomes included time to symptom onset, technical success rate, changes in QoL, and pre-procedure to post-procedure body weight variation.

## Patients and methods

### Study population

A retrospective case series review was conducted, including all patients diagnosed with axis deviation following sleeve gastrectomy who subsequently underwent endoscopic stricturotomy at the Hospital das Clínicas, Faculty of Medicine, University of São Paulo (HCFMUSP), between 2019 and 2024. Eligible patients were those with clinically significant symptoms of gastric axis torsion, confirmed by both endoscopic and radiologic assessments, in whom conservative management or isolated dilation was deemed inappropriate or had already failed. No formal changes were made to the institutional patient selection criteria during the study period.

### Data collection and analysis

Clinical data were obtained via systematic review of electronic medical records and follow-up telephone interviews. The objective was to evaluate effectiveness of endoscopic stricturotomy in alleviating symptoms associated with functional gastric outlet obstruction using a combination of clinical, anatomical, and functional parameters.

Considering that post-sleeve gastrectomy axis deviation may clinically resemble gastroparesis, symptom severity was assessed using the Gastroparesis Cardinal Symptom Index (GCSI), a validated subscale of the Patient Assessment of Upper Gastrointestinal Disorders Symptom Severity Index (PAGI-SYM), which quantifies upper gastrointestinal symptoms typically associated with motility disorders.

During follow-up interviews, patients rated frequency and severity of nausea/vomiting, early satiety, and abdominal bloating using a 6-point Likert scale (0 = none; 5 = very severe). The composite GCSI score was used to categorize overall symptom severity and to quantify clinical response by comparing pre-procedure and post-procedure values ( Table 1 ).

**Table:** **Table 1** Gastroparesis Cardinal Symptom Index (GCSI).

**Symptom subscale**	**Symptom**	**None**	**Mild**	**Moderate**	**Moderately severe**	**Severe**	**Very severe**
**Nausea/vomiting**	Nausea	0	1	2	3	4	5
	Retching	0	1	2	3	4	5
	Vomiting	0	1	2	3	4	5
**Early satiety**	Stomach fullness	0	1	2	3	4	5
	Inability to finish a normal-sized meal	0	1	2	3	4	5
	Postprandial fullness	0	1	2	3	4	5
	Loss of appetite	0	1	2	3	4	5
**Bloating**	Abdominal bloating	0	1	2	3	4	5
	Abdomen visibly larger	0	1	2	3	4	5
GCSI, Gastroparesis Cardinal Symptom Index.							

Patients were also asked to subjectively assess their overall QoL using a visual analog scale (VAS) ranging from 0 (no QoL) to 10 (excellent QoL), administered both before and after the procedure.

Pre-gastrectomy (baseline), pre-stricturotomy (onset of symptoms), and post-stricturotomy (resolution/last follow-up) weights were obtained from patient self-report during telephone interviews and confirmed when available in medical records. Because self-reported data were used, this was clearly noted and considered a potential source of bias in the study limitations.

### Contrast radiographic studies (esophagogastroduodenal/barium series)

These studies were performed according to institutional protocol with patients in supine and upright positions after barium ingestion. Fluoroscopic images were obtained to assess contrast column and passage dynamics. Reports were reviewed by a radiologist, focusing on retention pattern, transit speed, axis deviation/angulation, and focal narrowing. All exams were jointly reviewed by the endoscopist and radiologist to standardize findings and classify axis deviation/torsion versus focal stenosis.

### Assessment of luminal narrowing

Endoscopy was performed for qualitative evaluation of scope passage (free, resistant, or impossible); when feasible, approximate caliber was described based on gastroscope/probe passage.

Radiology was performed for qualitative grading of obstruction (mild/moderate/severe delay, retention, or deviation); objective luminal diameter measurement was not routinely performed (limitation).

### Classification applied in this study

Diagnosis required compatible symptoms plus endoscopic/radiological evidence of deviation or stenosis. Cases were categorized as “predominant torsion/angulation” or “predominant focal stenosis.”

Finally, technical success was defined as completion of the stricturotomy without conversion or intra-procedural AEs were also documented.

### Technical description

#### Endoscopic stricturotomy procedure

All procedures were performed under general anesthesia with CO₂ insufflation. After diagnostic endoscopy and therapeutic planning, submucosal injection was made at the proximal mucosa near the staple line, followed by a longitudinal incision and creation of a submucosal tunnel toward the angulated or tortuous segment. Selective myotomy of the involved muscular fibers was carried out to reduce angulation and increase gastric compliance. Hemostasis was achieved as required, and luminal patency was confirmed. Mucosal closure was not routinely necessary, but clips or sutures were applied when indicated; in selected high-risk cases, prophylactic vacuum therapy or drainage was considered.

#### Mechanism of action

Endoscopic stricturotomy relieves obstruction caused by gastric axis deviation by dividing the responsible muscle fibers, thereby correcting angulation and restoring forward passage without resecting fibrotic tissue.

#### Comparison with conventional methods

Balloon dilation expands focal fibrotic strictures by radial force but often requires repeated sessions, carries a risk of perforation, and does not correct angulation. SEMS maintain patency through radial pressure and are used when dilation fails, but their utility is limited by migration, discomfort, and perforation risk. Compared with these methods, endoscopic stricturotomy directly addresses axis deviation and compliance, offering a minimally invasive and targeted approach to angulation-related obstruction.

### Statistical analysis

Data analyses were performed using R software, version 4.4.2. The Shapiro-Wilk test was applied to assess normality of distribution of continuous variables. For paired comparisons, the paired *t* -test was used for normally distributed data, whereas the Wilcoxon signed-rank test was employed for non-normally distributed data. *P* < 0.05 was considered statistically significant ( **Supplementary Table 1** ).

### Inclusion and exclusion criteria

Patients with symptomatic obstruction (nausea, vomiting, oral intolerance, weight loss, or dysphagia) and endoscopic/radiologic evidence of gastric axis torsion or tortuous stenosis were included if conservative measures failed, anatomy was favorable (angulation > fibrosis), and clinical stability allowed endoscopy under general anesthesia. Mild torsion without symptoms was managed conservatively, whereas purely fibrotic strictures underwent dilation or revisional surgery.

Exclusion criteria comprised non-gastric strictures, complete gastric torsion, and incomplete medical records. Non-gastric strictures (e.g., primary esophageal or post-surgical intestinal lesions) were excluded due to their distinct pathogenesis, anatomy, and management, which would reduce cohort homogeneity and confound stricturotomy outcomes.

## Results

### Data analysis

Eight patients were included in the final analysis after exclusions: one due to gastric axis torsion from distal esophageal cancer and two for incomplete symptom data. The cohort had a mean age of 53 years and was predominantly female (5 women, 3 men). One patient had previously undergone balloon dilation for post-sleeve gastrectomy stricture.

Endoscopic stricturotomy was performed, on average, 31.3 months after sleeve gastrectomy. All procedures were conducted under general anesthesia by an experienced interventional endoscopist, with anesthesiology support.

Changes in patient body weight were assessed at three distinct timepoints: pre-bariatric surgery, post-sleeve gastrectomy at the onset of stricture-related symptoms, and post-stricturotomy following symptom resolution. Baseline patient characteristics and mean body weights at each stage were calculated and are presented in Table 2 and Table 3 .

**Table:** **Table 2** Patient characteristics and weight variation.

**Baseline characteristics**						**Weight variation (kg)**		
**Age**	**Gender**	**Surgery**	**Stricturotomy**	**GCSI pre**	**GCSI post**	**Before sleeve**	**Before stricturotomy**	**Stricturotomy**
53	F	2023	09/2023	29	16	108,6	76	74.6
70	M	2020	07/2023	32	18	113	78	73
69	M	2015	06/2024	35	4	130	75	86
54	F	2023	03/2023	23	6	105	85	75
40	M	2022	09/2022	23	16	100	70	70
49	F	2019	04/2020	34	30	102	72	72
37	F	2019	10/2020	33	9	107	66	66
54	F	2017	10/2019	34	15	108	86	77
GCSI, Gastroparesis Cardinal Symptom Index; Pre, before stricturotomy; Post, after stricturotomy.								

**Table:** **Table 3** Mean body weight before sleeve gastrectomy, before stricturotomy, and after stricturotomy.

**Period**	**Mean weight (kg)**
**Before sleeve gastrectomy**	109.2
**Before stricturotomy**	76
**After stricturotomy**	74.2

GCSI was applied using a 0 to 5 Likert scale, grouped into subscales (nausea/vomiting, early satiety, bloating) and summed for a global score. QoL was measured on a 0 to 10 VAS (0 = worst, 10 = best). Pre- and post-procedure means were reported (3 → 7.75) with paired statistical analysis.

With regard to symptoms associated with functional gastric axis stenosis, a significant reduction in symptom severity was observed across all domains of GCSI. Mean scores across all subscales demonstrated a consistent downward trend.

Mean GCSI item scores (pre- vs. post-intervention) were presented with *P* values. Significant improvements ( *P* < 0.05) were observed for nausea ( *P* = 0.04), retching ( *P* = 0.01), bloating ( *P* = 0.02), and satiety ( *P* = 0.008–0.03). For clinical interpretation, we also analyzed the percentage reduction of symptoms rated as “moderate,” “severe,” or “very severe” before and after the procedure. All results are presented in Table 4 .

**Table:** **Table 4** Comparison of nausea/vomiting, early satiety and abdominal distension symptoms before and after stricturotomy.

	**Nausea**		**Retching**		**Vomiting**		**Stomach fullness**		**Unable to finish a normal-size meal**		**Feeling excessively full after meals**		**Appetite loss**		**Bloating**		**Stomach or belly visibly larger**	
**Severity**	**Before**	**After**	**Before**	**After**	**Before**	**After**	**Before**	**After**	**Before**	**After**	**Before**	**After**	**Before**	**After**	**Before**	**After**	**Before**	**After**
**None**	1 (12.5%)	0 (0%)	0 (0%)	0 (0%)	1 (12.5%)	4 (50.0%)	0 (0%)	1 (12.5%)	0 (0%)	1 (12.5%)	0 (0%)	2 (25.0%)	2 (25.0%)	3 (37.5%)	0 (0%)	2 (25.0%)	0 (0%)	2 (25.0%)
**Very mild**	0 (0%)	3 (37.5%)	0 (0%)	3 (37.5%)	2 (25.0%)	2 (25.0%)	0 (0%)	3 (37.5%)	0 (0%)	2 (25.0%)	0 (0%)	2 (25.0%)	1 (12.5%)	1 (12.5%)	0 (0%)	4 (50.0%)	0 (0%)	4 (50.0%)
**Mild**	1 (12.5%)	4 (50.0%)	2 (25.0%)	3 (37.5%)	2 (25.0%)	1 (12.5%)	2 (25.0%)	2 (25.0%)	0 (0%)	2 (25.0%)	0 (0%)	2 (25.0%)	1 (12.5%)	3 (37.5%)	0 (0%)	1 (12.5%)	3 (37.5%)	1 (12.5%)
**Moderate**	3 (37.5%)	1 (12.5%)	4 (50.0%)	2 (25.0%)	1 (12.5%)	1 (12.5%)	1 (12.5%)	1 (12.5%)	3 (37.5%)	2 (25.0%)	4 (50.0%)	1 (12.5%)	3 (37.5%)	1 (12.5%)	3 (37.5%)	0 (0%)	2 (25.0%)	0 (0%)
**Severe**	2 (25.0%)	0 (0%)	1 (12.5%)	0 (0%)	1 (12.5%)	0 (0%)	2 (25.0%)	0 (0%)	1 (12.5%)	0 (0%)	1 (12.5%)	0 (0%)	0 (0%)	0 (0%)	1 (12.5%)	0 (0%)	0 (0%)	0 (0%)
**Very severe**	1 (12.5%)	0 (0%)	1 (12.5%)	0 (0%)	1 (12.5%)	0 (0%)	3 (37.5%)	1 (12.5%)	4 (50.0%)	1 (12.5%)	3 (37.5%)	1 (12.5%)	1 (12.5%)	0 (0%)	4 (50.0%)	1 (12.5%)	3 (37.5%)	1 (12.5%)

Symptom changes before and after stricturotomy were analyzed using both statistical significance and effect size. For normally distributed variables, including nausea, retching, gastric fullness, loss of appetite, and global GCSI score, paired *t* -tests and 95% confidence intervals (Cis) confirmed significant improvements and provided estimates of the plausible range of change, highlighting the clinical relevance of the intervention. Non-normally distributed variables, such as vomiting, inability to finish a normal-sized meal, postprandial bloating, abdominal distension, and visibly larger abdomen, were analyzed using Wilcoxon signed-rank tests, with median differences showing consistent trends toward symptom reduction. Overall, the results are presented in the supplementary material, and indicate that stricturotomy produces meaningful improvements in gastroparesis symptoms, with CIs quantifying magnitude of benefit for normally distributed outcomes.

Clinical success was defined as significant improvement in symptoms, measured by reduction in global GCSI score and/or self-reported improvement on the analog QoL scale, with adequate oral intake without need for long-term enteral nutrition, up to the last documented follow-up.

### Overall clinical response

Global GCSI score, derived from the sum of the three symptom domains, significantly decreased from 35.44 pre-procedure to 21.66 post-stricturotomy ( *P* = 0.014). This reduction underscores the overall effectiveness of endoscopic stricturotomy in alleviating symptoms of functional gastric axis stenosis and reinforces its role as a minimally invasive therapeutic option ( Table 5 ).

**Table:** **Table 5** Comparison of overall average Gastroparesis Cardinal Symptom Index (GCSI) before and after stricturotomy.

	**Overall average GCS** I
**Average GCSI before stricturotomy**	35,44
**Average GCSI after stricturotomy**	21,66
GCSI, Gastroparesis Cardinal Symptom Index.	

Patients were also asked to assess their perceived QoL using a VAS administered both before and after endoscopic stricturotomy. The scale ranged from 0, indicating no QoL, to 10, representing excellent QoL. Changes in individual and average VAS scores are summarized in Table 6 and Table 7 .

**Table:** **Table 6** Patient-reported quality of life scores before and after stricturotomy.

**Patient**	**Quality of life before stricturotomy**	**Quality of life after stricturotomy**
1	3/10	9/10
2	4/10	8/10
3	3/10	8/10
4	3/10	9/10
5	3/10	7/10
6	4/10	6/10
7	2/10	7/10
8	2/10	8/10

**Table:** **Table 7** Average grade applied to quality of life before and after stricturotomy.

**Period**	**Average grade applied to quality of life**
Before stricturotomy	3/10
After stricturotomy	7.75/10

One patient did not experience significant symptomatic improvement: a 49-year-old woman with a history of sleeve gastrectomy (2019), hiatal hernia, and gastroesophageal reflux disease. Her weight decreased from 102 kg pre-sleeve to 72 kg post-sleeve and remained 72 kg after stricturotomy. QoL improved modestly from 4 to 6. Prior interventions included endoscopic dilation to 30 mm with placement of a partially covered metal stent and nasoenteric tube (05/2020). One month later, the nasoenteric tube was removed, and a fully covered SEMS was placed over the initial stent to further optimize symptom control.

### Adverse events

Intraoperative pneumoperitoneum occurred in two patients and was managed successfully with endoscopic decompression. In two other cases, endoscopic vacuum therapy (EVT) was applied for mucosal hypoperfusion, detected by pale mucosal appearance, absence of capillary bleeding, and presence of whitish areas post-myotomy. Patients remained hospitalized with a modified vacuum at 125 mm Hg for 24 hours and removal was guided by endoscopic mucosal assessment. EVT promotes healing by enhancing tissue perfusion, angiogenesis, and cellular proliferation, and controlling exudate and bacterial load.

EVT carries potential risks such as mucosal injury, bleeding, or infection. Careful patient selection, close monitoring, and standardized protocols are essential to minimize these risks, and future prospective studies are needed to establish optimal parameters and confirm the safety and efficacy of this approach.

One patient was readmitted 2 days after discharge following stricturotomy with persistent abdominal pain, mild tachycardia, and subcutaneous emphysema. Contrast-enhanced computed tomography (CT) revealed a post-stricturotomy gastric fistula, free intraperitoneal air, and extensive pneumomediastinum. Early recognition enabled prompt multidisciplinary management, including percutaneous drainage and EVT, ultimately resulting in full clinical resolution.

### Imaging studies

Pre-procedure endoscopy consistently demonstrated significant gastric axis deviation with acute angulation of the lumen, resulting in resistance to the passage of a standard-caliber endoscope.

Following stricturotomy, endoscopic evaluation showed marked improvement in luminal alignment, facilitating smooth advancement into the distal stomach.

**Fig. 1:**
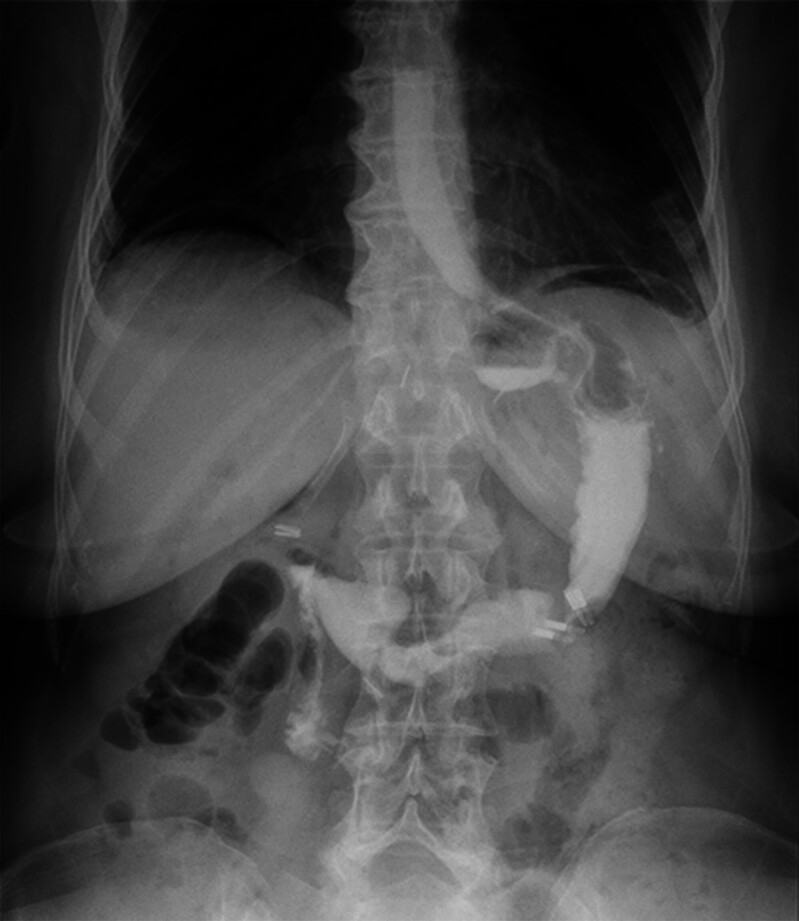
Contrast retention causing delayed gastric emptying. Source: Electronic medical record.

**Fig. 2:**
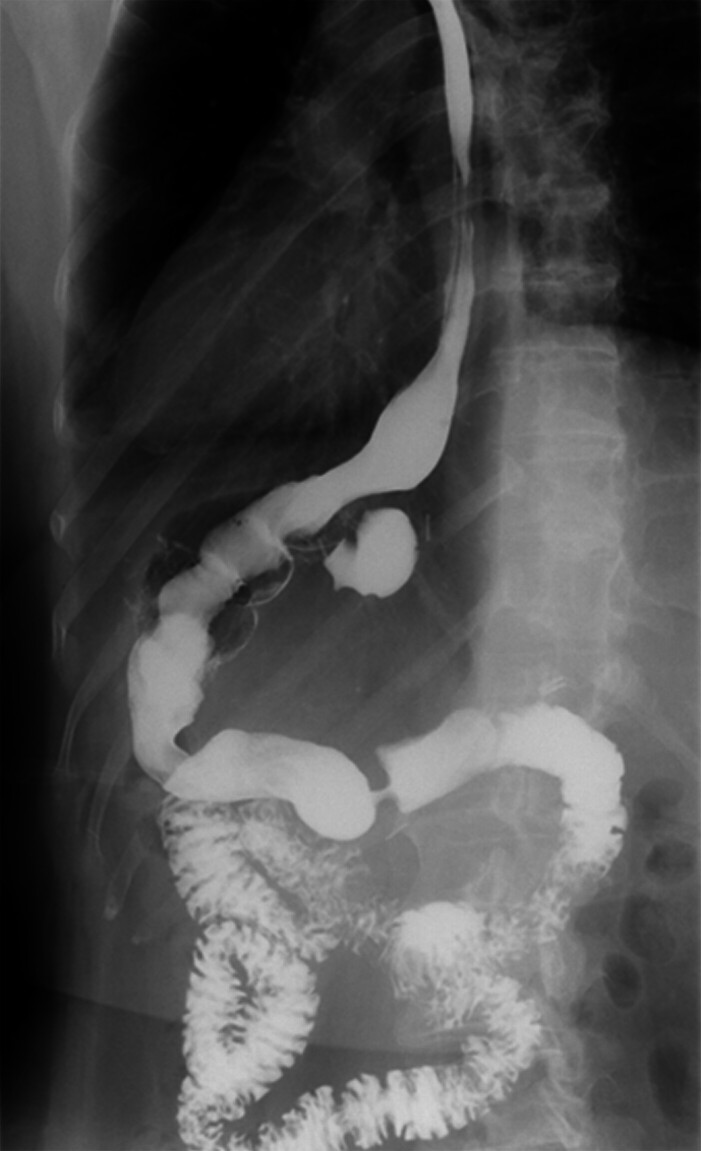
Luminal narrowing without hindrance to contrast progression. Source: Electronic medical record.

Similarly, pre-intervention upper gastrointestinal series revealed contrast retention and delayed gastric emptying, findings consistent with functional gastric outflow obstruction ( Fig. 1 ). Post-procedure imaging demonstrated restored gastric transit with prompt contrast progression into the duodenal loops ( Fig. 2 ).

## Discussion

Helical stenosis after sleeve gastrectomy affects 0.7% to 4% of patients and is often caused by torsion of the gastric sleeve, leading to functional obstruction. Conventional endoscopic treatments, including balloon dilation and SEMS, have limited efficacy, may require multiple interventions, and carry risks such as stent migration or perforation 4 5 .

In this setting, endoscopic stricturotomy represents a minimally invasive and technically feasible alternative. Through submucosal tunneling, the procedure enables anatomical realignment of the gastric axis and restoration of luminal patency, resulting in effective symptom relief and improved health-related QoL 6 .

In our case series, a consistent reduction in symptom severity was observed across all domains of the GCSI. Statistically significant improvements were recorded in nausea, retching, gastric fullness, and abdominal bloating, reflecting the therapeutic efficacy of stricturotomy in managing functional gastric axis stenosis.

The decrease in the global GCSI mean score following stricturotomy further reinforces the effectiveness of the procedure. Pre- and post-procedure score analysis revealed a statistically significant difference, demonstrating that the technique is effective in reducing stenosis-related symptoms and relieving gastric obstruction, offering durable clinical benefits.

The significant decrease in the global mean GCSI score further underscores the clinical benefit of this intervention. This change highlights not only relief of mechanical obstruction but also durability of symptom control, positioning stricturotomy as a viable alternative to conventional endoscopic or surgical management strategies; however, the team learning curve and increasing familiarity with the technique may have influenced outcomes, representing a potential temporal bias.

QoL is markedly compromised in patients with post-sleeve gastrectomy gastric stenosis. Prior studies have demonstrated the substantial impact on daily activities, eating habits, and emotional well-being. These manifestations may lead to malnutrition, weight loss, fatigue, social withdrawal, and emotional distress. The GCSI has been validated as a sensitive instrument to evaluate symptom burden in this population, with scores > 20 indicative of significant QoL impairment 7 .

In this study, the reduction in the mean GCSI score from 35.44 to 21.66, alongside the increase in self-reported VAS QoL scores from 3.0 to 7.75, demonstrates a clinically relevant improvement following stricturotomy. These findings reinforce the notion that endoscopic stricturotomy not only alleviates anatomical obstruction but also translates into meaningful improvements in patient functional status and overall well-being.

Postoperative weight loss following sleeve gastrectomy is influenced by a variety of individual factors, including age, sex, preoperative body mass index, and adherence to postoperative dietary and physical activity recommendations 8 . On average, a weight reduction of 10% to 12% is expected within the first month, and 15% to 25% within the first 3 months after surgery.

In the cohort evaluated in this study, mean weight loss following vertical sleeve gastrectomy was approximately 33.19%, with a decrease in mean body weight from 109.2 kg to 76 kg. This reduction exceeds typical postoperative expectations and may be attributed, at least in part, to feeding intolerance and nutritional compromise secondary to gastric axis torsion.

Although laboratory markers (proteins, vitamins, minerals) were not obtained for nutritional monitoring, post-stricturotomy dietary reintroduction was associated with an average weight stabilization of 2%. This modest decline reflects symptom resolution and nutritional reestablishment, supporting clinical efficacy of the procedure in restoring normal gastrointestinal function. However, longer follow-up is needed to confirm full nutritional recovery.

As for potential AEs, endoscopic stricturotomy carries inherent risks, including perforation, bleeding, and localized infection. Reported complication rates vary across studies. In a large series, the perforation rate was 0.4% per procedure and the bleeding rate 3.3%, both successfully managed endoscopically 9 . Subsequent studies have reported slightly higher complication rates, with AEs observed in 6.9% of procedures 10 and bleeding requiring transfusion in 14.3% of cases 11 . A systematic review summarized these findings, noting that although such events are uncommon, they may occasionally require prompt intervention 12 .

In this series, one patient evaluated 1 year after stricturotomy reported persistent symptoms and required reintervention with a SEMS. Two patients developed intraoperative pneumoperitoneum, both successfully managed endoscopically without surgery. One additional patient experienced pneumoperitoneum with extensive pneumomediastinum within 24 hours post-procedure, presenting with abdominal pain, mild tachycardia, and subcutaneous emphysema; CT revealed free intraperitoneal air, mediastinal emphysema, and extraluminal contrast leakage. Prompt recognition enabled percutaneous drainage and EVT, resulting in gradual improvement and full recovery.

AEs were mostly related to procedure complexity: pneumoperitoneum from CO₂ leakage during submucosal dissection and gastric fistula from multifactorial causes including myotomy depth, local hypoperfusion, and tissue fragility. Preventive measures—CO₂ insufflation, careful tunneling, strict hemostasis, prophylactic EVT in high-risk cases, and multidisciplinary early management—were key to avoiding severe outcomes. With refined technique, structured training, and careful patient selection, the safety profile of endoscopic stricturotomy can be further improved.

At our institution, prophylactic EVT is routinely employed when mucosal or submucosal hypoperfusion is identified during the procedure, typically due to mechanical trauma from myotomy. The rationale for this approach is to stimulate neoangiogenesis, enhance tissue healing, and prevent delayed AEs.

Among other endoscopic therapies for post-sleeve gastrectomy stenosis, balloon dilation has been reported to carry an overall AE rate of approximately 2.5%, with perforation occurring in about 0.7% of cases 12 . Gastric stents are associated with higher rates of stent migration, which may occur in up to 7% of cases. In addition, patients often report significant discomfort or pain, and there remains a risk of perforation, particularly in anatomically complex cases 13 .

Although endoscopic stricturotomy has demonstrated favorable efficacy for managing postoperative gastric axis stenosis, it is a technically advanced procedure that necessitates a highly experienced endoscopy team and availability of specialized infrastructure to address potential treatment-related events. Prompt recognition and management of AEs—such as pneumoperitoneum, tissue hypoperfusion, and fistula formation—is critical to ensure patient safety and achieve optimal outcomes.

To mitigate bias, we prioritized medical records for objective variables (exams, procedures, hospital notes). Telephone interviews were used only to complement missing data (e.g., retrospective GCSI scores, unrecorded weight), always with a validated instrument (GCSI). To reduce recall bias, we applied structured questions, cross-checked self-reports with prior records when possible, and explicitly noted self-reported data. Despite these measures, some risk of bias remains due to the retrospective design and reliance on subjective recall, which we acknowledged as a study limitation and suggested prospective standardized assessments for future research.

Despite the promising results, this study has several limitations. Most notably, the small sample size (n = 8) restricts statistical power, increasing the risk of type II error and limiting the ability to detect small or moderate effects. Although statistically significant improvements were observed (e.g., global GCSI score), these findings should be interpreted with caution, given the small cohort size. Larger, preferably prospective and multicenter studies are needed to confirm the magnitude of effect and provide more reliable estimates of complication and recurrence rates.

Selection bias may also be present because cases were retrospectively identified from a referral center, where patients more often have severe conditions or have access to specialized care, limiting generalizability. Information bias is another concern because data were partly derived from chart review and patient self-report during telephone interviews, particularly for weight at certain time points. Although validated instruments such as the GCSI were used, measurement errors and recall bias cannot be excluded.

In addition, absence of a control group prevented adjustment for potential confounders (e.g., time since bariatric surgery, prior interventions, comorbidities), which could have influenced outcomes. Another limitation was the lack of standardized contrast radiographic studies of all patients, potentially affecting consistency in anatomical assessment.

Taken together, these limitations underscore that our findings primarily suggest a signal of efficacy and acceptable safety in an experienced center but do not replace higher-level evidence. Prospective studies with larger cohorts, standardized imaging protocols, control groups, and longer follow-up to evaluate stricture recurrence, reflux, and late symptoms with follow-up beyond 2 to 3 years are required to validate these results and define the true role of endoscopic stricturotomy.

## Conclusions

Endoscopic stricturotomy is an effective and safe alternative for treatment of gastric stenosis following vertical sleeve gastrectomy, providing relief of gastric obstruction symptoms and significant improvement in patient QoL.

## Supplementary Material

Supplementary Material Zusatzmaterial
