# Supplementary material for: Endoscopic stricturotomy for axis-related gastric outlet obstruction following sleeve gastrectomy
Source: Endosc Int Open. 2025 Dec 3;13:a27433189. doi: 10.1055/a-2743-3189 (PMC12704506; doi:10.1055/a-2743-3189)
Supplement: Supplementary file 1 — Supplementary Material [file 10-1055-a-2743-3189_27500405.pdf]

Thieme

Supplementary Table 1 Statistical significance analysis of scores obtained in all GCSI domains.

Variable	Shapiro-Wilk *P* value (before)	Shapiro-Wilk *P* value (after)	Wilcoxon *P* value	Paired *t*-test *P* value	Difference (mean or median)	95% CI low	95% CI high
Nausea	0.4158	0.05552	0.04007	-	1.25	-	-
Retching	0.156	0.06724	–	0.01898	1.25	0.44	2.06
Vomiting	0.8006	0.03563	0.0875	–	1.5	-	-
Gastric fullness	0.08159	0.2686	–	0.008309	1.88	0.86	2.89
Inability to finish a normal-sized meal	0.005711	0.7314	0.03103	–	2.0	-	-
Postprandial bloating	0.005711	0.3342	0.03552	–	2.0	-	-
Loss of appetite	0.425	0.1139	–	0.02094	0.875	0.30	1.45
Abdominal distension	0.005711	0.007872	0.0213	–	2.5	-	-
Visibly larger abdomen	0.01735	0.007872	0.0213	–	1.5	-	-

CI, confidence interval; GCSI, Gastroparesis Cardinal Symptom Index.
